# Endemic Diseases: Globalization, Urbanization, and Immunosuppression

**DOI:** 10.1155/2013/390986

**Published:** 2013-04-07

**Authors:** Maria Aparecida Shikanai Yasuda, Pedro Albajar Viñas

**Affiliations:** ^1^Department of Infectious and Parasitic Disease, Faculdade de Medicina, University of Sao Paulo (USP), São Paulo, SP, Brazil; ^2^Laboratorio de Imunologia, Avenida Enéias de Carvalho Aguiar, 500 Térreo, Sala 4, 05403-000 Sao Paulo, SP, Brazil; ^3^HIV/AIDS, Tuberculosis, Malaria and Neglected Tropical Diseases (HTM), Innovative and Intensified Disease Management (IDM), World Health Organization, Geneva, Switzerland

Globalization and urbanization of endemic diseases represent major challenges in developed and underdeveloped countries. Massive migration within and between the countries was registered particularly since the beginning of this century. As intrinsic part of human history, migration has increased both in number and speed due to economic crises, civil war, and natural disasters. As consequence of the coexistence of transmissible and nontransmissible diseases in urban and periurban centers, several noninfectious chronic diseases occur in association with infectious diseases, contributing synergistically to increase the morbidity and mortality of diseases in urban centers.

In Latin America, the migration from rural area to great cities was registered in the context of socioeconomical disparities and poor basic sanitary infrastructure and low access to preventive medicine. In developed countries, vulnerable groups of migrants have less access to preventive medicine and to health care system bringing new potential for transmission of these diseases by alternative routes. 

The globalization of human Chagas disease around the world is discussed in the context of bioecological, sociocultural, and political aspects, including relevant topics as migration and human Chagas disease, international flows to nonendemic countries, vectors and reservoirs movements, Role of the remaining sylvatic cycle *of T. cruzi*, and medical management Chagas disease in a globalised world as a very critical point. 

The urbanization of tropical diseases such as malaria and leishmaniasis has been reported in two articles about urban malaria transmission in sub-Saharan Africa and periurban malaria in Lusaka, Zambia and other about canine visceral leishmaniasis in an urban area in Brazil”.

New diagnostic methods for dengue virus and the expression of new immunoregulatory molecules by *Trypanosoma cruzi *SSP4 amastigote protein were also discussed in this special number.

In parallel to alternative forms of transmission, the reactivation of neglected tropical diseases under immunosuppressive conditions (HIV infection, immunosuppressive therapy, cytotoxic treatment for cancer and autoimmune diseases, immunobiological drugs for autoimmune disease, transplantation, and graft rejection) represents new challenges in urban centers. The experience in the prophylaxis of *Toxoplasma gondii *myocarditis in heart transplantation is presented and the influence of gender and age in cysticercosis was reported in a case control study.

Finally, in this issue, two articles analyzed the mortality of HIV/AIDS as comorbidity of two tropical diseases: American visceral leishmaniasis and Chagas disease in Brazil.

*Maria Aparecida Shikanai-Yasuda* *Maria Aparecida Shikanai-Yasuda* *Pedro Albajar Viñas* *Pedro Albajar Viñas*

